# A Comparison of the Quality and Reliability of YouTube Videos Uploaded by Healthcare Professionals About Scoliosis in the Past Decade

**DOI:** 10.7759/cureus.44830

**Published:** 2023-09-07

**Authors:** Cigdem Cinar

**Affiliations:** 1 Department of Interventional Physiatry, Biruni University, Istanbul, TUR

**Keywords:** videos, youtube, scoliosis, gqs, discern score

## Abstract

Introduction: Internet-based medical education plays a crucial role in bolstering public health awareness and the competence of medical professionals; however, it must be dependable. We aimed to examine the quality and reliability of the videos uploaded to YouTube (YouTube, LLC, San Bruno, California, United States) by professional healthcare professionals about scoliosis from 2014 to 2023.

Methods: The study was performed between August 1 and August 15, 2023. The keywords 'scoliosis', 'scoliosis-kyphosis brace', 'scoliosis exercises', 'scoliosis surgeries', and 'scoliosis-kyphosis exercises' were searched on YouTube. Only YouTube videos uploaded by healthcare professionals between January 1, 2014, and July 31, 2023, and only videos in the English language were examined. Video characteristics were evaluated and recorded for all videos. The quality and reliability of videos were assessed with two different questionnaire scales including the modified DISCERN score and the Global Quality Score (GQS).

Results: The mean number of views per video was 3778 in videos uploaded between 2014 and 2018, and 3120 in videos uploaded between 2019 and 2023 (p= 0.004). Video length and number of likes were statistically significantly lower in the videos of the last five years (p=0.001, and p=0.001). Thirty-one percent of the professional videos uploaded between 2014 and 2018 were directed at healthcare professionals. This rate was 12% in the last five years and was statistically significantly lower (p=0.001). The DISCERN score average was 3.7 between 2014 and 2018 and it was 3.2 between 2019 and 2023 (p=0.001). Similarly, the mean GQS was statistically significantly higher in the last five years compared to the five years prior (3.8 vs. 3.3, p=0.001).

Conclusion: The present study determined that the quality and reliability of YouTube videos about scoliosis significantly increased in the last five years compared to the previous five years with a significant decrease in video duration. Also, among videos uploaded in the last five years, the number of videos for the patient was significantly higher. In contrast, the ‘like’ and ‘view’ numbers were significantly lower in YouTube videos about scoliosis in the last five years.

## Introduction

Scoliosis is simply defined as a deformity consisting of abnormal lateral curvature and rotation of the vertebrae and may be idiopathic or secondary to hereditary diseases, neuromuscular pathologies, or trauma [1]. The true prevalence of scoliosis is unknown, as there are no globally accepted screening methods [2]. Adobor et al. examined 4000 Norwegian children under 12 years of age in 2011 and found that the prevalence of curvature greater than 10° was 0.55% and the prevalence of curvature greater than 20° was 0.13% [3]. In a systemic review by Altaf et al., the prevalence of curves greater than 10° was found to be 1.1%, and the prevalence of curves greater than 20° was 0.2% [4]. These rates show that scoliosis is a serious and common public health problem. On the other hand, people have been using social media platforms increasingly in recent years to get information about their health problems [5]. Easy access to sources, free of charge, and access to information from many sources can be counted as the most important reasons for society to use social media applications on health-related issues [6].

YouTube (YouTube, LLC, San Bruno, California, United States) is the most used social media application with approximately three billion users in 120 countries [7]. Freeman et al., in their study in which they examined the effect of source style on people, showed that visual sources were more interesting than audio-only or written-only sources [8]. While the YouTube platform currently contains millions of videos [9], hundreds of new videos on different topics are added every day. However, YouTube does not have strict criteria for uploading videos, and videos containing incorrect or obscure information may be uploaded too. Ergul demonstrated that numerous YouTube videos about uterine leiomyoma contained misleading data, despite the high ratings of the videos [10]. Similarly, Cetin et al. analyzed the quality of professional and non-professional YouTube videos about coronary artery bypass grafting and showed the insufficient reliability and quality of these videos [11]. Additionally, they stated that videos that were uploaded by professional healthcare workers had better quality and reliability.

Although previous studies have examined the quality of YouTube videos on different medical conditions based on different factors (duration, upload source, likes, etc.), to our knowledge, there has been no study involving the evolution of YouTube videos uploaded by healthcare professionals. In this study, we aimed to examine the quality and reliability of the videos uploaded on YouTube by healthcare professionals about scoliosis in the past decade.

## Materials and methods

This study was carried out between August 1 and August 15, 2023. An experienced physical therapist watched each included video twice in the two-week period. The keywords 'scoliosis', 'scoliosis-kyphosis brace', 'scoliosis exercises', 'scoliosis surgeries', and 'scoliosis-kyphosis exercises' were searched on YouTube and sorted by relevance, and a playlist containing these videos was created.

Inclusion and exclusion criteria

Only videos uploaded by healthcare professionals (physiotherapists or orthopedic surgeons) from January 1, 2014, to July 31, 2023, and only YouTube videos in the English language were examined. Videos prepared in a language other than English, self-promotional videos, silent videos, re-uploaded videos, and videos with content unrelated to the research subject were excluded. Moreover, videos shorter than two minutes and longer than 15 minutes were not included in the study. Lastly, videos of news channels and videos containing the experiences of patients or their relatives were excluded from the study.

Process of evaluation

A physical therapy specialist with eight years of experience in physiotherapy and scoliosis analyzed all the videos. Video characteristics were evaluated and recorded for all videos. The number of views, number of ‘likes’ and ‘dislikes’, number of comments number, and length of the video were noted. Also, the video target group was divided into healthcare professionals and patients. The quality and reliability of videos were assessed with two different questionnaire scales (modified DISCERN score and Global Quality Score (GQS)).

Modified DISCERN Score and GQS

The modified DISCERN questionnaire was determined to evaluate the quality of written or visual content, and the questionnaire included five questions. Each question answered ‘yes’ (1 point) or ‘no’ (0 point). Five points indicated the highest quality [12]. GQS is developed to analyze the quality and usability of videos, and the GQS questionnaire is scored from 1 point to 5 points. A score of 1 indicates low quality and a score of 5 indicates high quality [13]. In order to evaluate the quality of professional YouTube videos about scoliosis over the years, the videos uploaded from 2014 to 2023 were divided into two groups: Group 1 with videos from January 1, 2014, to December 31, 2018, and (ii) Group 2, with videos uploaded from January 1, 2019, to July 31, 2023. The 100 most watched videos that met the study criteria were selected from both groups. These videos were compared according to their video characters, modified DISCERN score, and GQS.

Statistically analysis

IBM SPSS Statistics for Windows, Version 26.0 (Released 2019; IBM Corp., Armonk, New York, United States) was used. The Kolmogorov-Smirnov test was done to check the normal distribution of the parameters. The Independent Student's t-test was done for comparison of the continuous parameters. Categorical variables were compared using the χ2 test. The data were analyzed at 95% confidence level (CI), and a p-value <0.05 was accepted as statistically significant.

## Results

**Figure 1:**
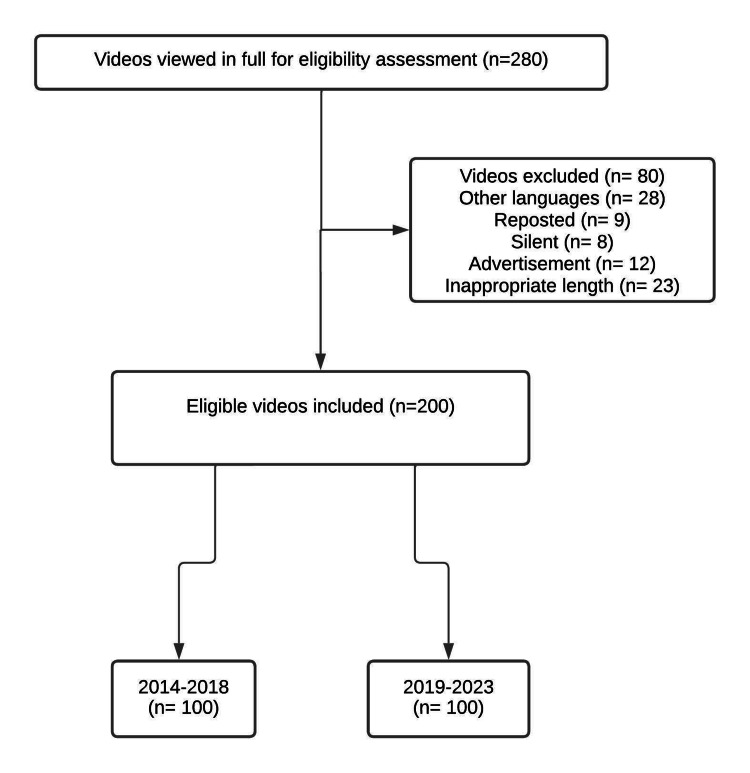
Flow chart

The flowchart of the study is given in Figure 1. A total of 280 videos were first examined and 80 videos were excluded from the study (different languages, reposted videos, silent videos, advertisements, and inappropriate duration of videos). Finally, there were 100 videos in each group according to the study design.

The mean number of views per video was 3778 in the videos of Group 1, and 3120 in the videos in Group 2 (p= 0.004). Video length and number of likes were statistically significantly lower in the videos of Group 2 (p=0.001 and p=0.001, respectively). There was no statistically significant difference between the two groups in terms of dislikes and comments (p= 0.615 and p= 0.327, respectively). A total of 31% of the professional videos uploaded between 2014 and 2018 (Group 1) were directed at healthcare professionals. This rate was 12% in videos of Group 2, and was statistically significantly lower (p=0.001) (Table 1).

**Table 1:** Comparison of video features by categories

	2014-2018 (Group 1)	2019-2023 (Group 2)	p-value
Number of videos	100	100	
Video parameters, median (interquartile range)			
Number of views	3778 (2382-5175)	3120 (1464-4537)	0.004
Video length (min)	9 (5-14)	6 (3-9)	0.001
Likes	91 (56-140)	79 (44-125)	0.001
Dislikes	9 (4-13)	10 (4-14)	0.615
Comments	18 (7-30)	21 (7-34)	0.327
Target audience, n (%)			
Doctors or health workers	31 (31.0%)	12 (12.0%)	0.001
Patients	69 (69.0%)	88 (88.0%)	

**Figure 2:**
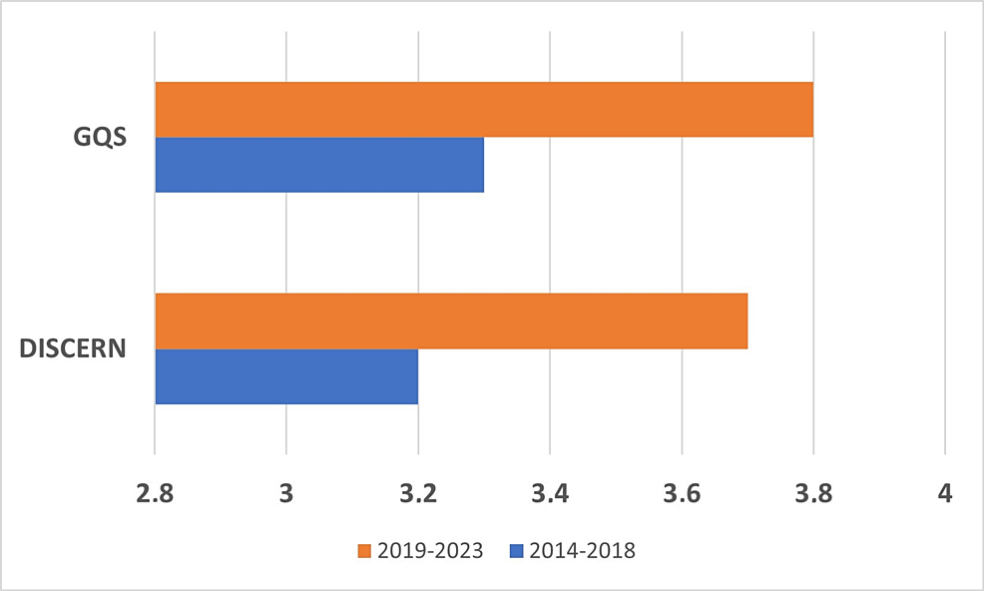
Comparison of DISCERN and GQS scores by years GQS: Global quality score

A comparison of DISCERN score and GQS scores between groups is shown in Figure 2. While the DISCERN score average was 3.7 in Group 2 (2019-2023), it was 3.2 in Group 1 (2014-2018) (p=0.001). Similarly, the mean GQS was statistically significantly higher in Group 2 compared to Group 1 (3.8 vs. 3.3, p=0.001).

## Discussion

Widespread use and easy access to digital resources have led many people to use social media to obtain information about diseases. Statistics about YouTube have shown that nine out of 10 internet users today prefer to use YouTube [14]. On the other hand, there are studies showing that YouTube videos uploaded by professionals, especially in the field of health, are of higher quality than non-professional sources [10,11]. However, there is no study in the literature showing the change in the quality of the videos uploaded by professional health workers over the years. Thus, we performed a study to analyze the quality of videos uploaded to YouTube by professional healthcare professionals about scoliosis in the last 10 years.

Our findings showed that the quality and reliability of YouTube videos about scoliosis significantly increased in the last five years compared to the five years prior to that. Moreover, video duration was significantly shorter in YouTube videos about scoliosis in the recent past five years. The improvement in the quality and reliability of YouTube videos about scoliosis over 2019 to 2023, along with shorter video durations, likely stems from a combination of factors, including advancements in medical knowledge, changes in YouTube algorithms, increased awareness, feedback from viewers, and a more competitive landscape among content creators. Also, among videos uploaded in 2019-2023, the number of videos directed at patients was significantly higher. This increase in the number of videos specifically created for patients in the last five years is likely a response to evolving healthcare trends that prioritize patient education, empowerment, and engagement. In contrast, the number of ‘likes’ and ‘views’ were significantly lower in YouTube videos about scoliosis in the last five years.

The modified DISCERN score and GQS were developed to assess the quality and reliability of information sources. Yuksel and Cakmak conducted a study to evaluate YouTube videos about coronavirus disease 2019 (COVID-19) and pregnancy, and while they emphasized the poor quality of uploaded videos, they also stated that videos uploaded by professional healthcare providers had significantly higher DISCERN scores, which indicated better quality and reliability [15]. Similarly, Ferhatoglu et al. reported significantly better DISCERN scores for YouTube videos uploaded by professional healthcare workers about bariatric surgery in comparison to non-professional videos [16]. Moreover, Kilinc and Sayar evaluated YouTube videos about orthodontics using GQS, and determined that the GQS was significantly better for videos uploaded by professional healthcare providers [17]. However, none of the aforementioned studies examined the evolution in the quality of videos uploaded from professional sources over time. For the first time, the current study demonstrated that the quality and reliability of YouTube videos about scoliosis by professional health workers significantly increased in the last five years compared to the five years prior to that.

The length of YouTube videos and their target audience can affect the number of views. In today's fast-paced digital environment, many viewers have shorter attention spans. Long-duration videos being too detailed can also be tiring for people. Kanber and Koseoglu analyzed the quality of YouTube videos on pediatric cardiac surgery anesthesia, but they did not find any significant difference in regard to video length and the target audience [18]. However, they had included both professional and non-professional videos in their study and they didn't compare videos by year. In the current study, findings revealed that YouTube videos uploaded about scoliosis from 2019 to 2023 were significantly shorter in duration, and among these videos, the number of videos directed at the patient was significantly higher. The observation that YouTube videos about scoliosis uploaded in the last five years were significantly shorter in duration implies that content creators have been condensing their information into more concise formats. Creators aim to deliver information efficiently and engage viewers who may have limited time or shorter attention spans. The finding that there is a significantly higher prevalence of videos intended for patients among those uploaded in the last five years suggests a shift in content creation priorities. Healthcare professionals and content creators appear to be recognizing the importance of catering to the needs of patients seeking information on scoliosis. 

YouTube video characteristics such as the number of likes and views are important to get more interaction. Sevgili and Baytaroglu did not find any significant difference in number of likes when comparing professional and non-professional YouTube videos about cardiac diseases [19]. Ergul found that the number of likes and the number of views were similar on YouTube videos on uterine leiomyomas for both groups [10]. In another study, Yuksel and Cakmak did not find any significant difference between professional and non-professional videos in regard to the number of likes, but they found that the number of views was significantly higher in professional videos [15]. In the present study, we found that the number of likes and the number of views were significantly higher in the videos uploaded to YouTube in the first five years (2014-2018); in our opinion, this is probably due to the longer existence of these videos on YouTube.

Limitations

This study is the first to analyze the quality of professional YouTube videos about scoliosis over 10 years, but the study contained only videos in the English language and this could be a limitation. However, English is the most used language on YouTube, and evaluation of more than one language could be confusing and difficult to analyze outcomes. Secondly, the present study analyzed a certain period, but new videos are constantly being shared on YouTube. In addition, the videos were evaluated by a physiotherapist with eight years of experience in physical therapy. Our data should be supported by different studies with a more experienced nd diverse team. Lastly, we searched for five keywords, and individuals can use different words beyond these five words when searching for videos on YouTube about scoliosis.

## Conclusions

The present study determined that the quality and reliability of YouTube videos about scoliosis significantly increased in the past five years compared to the five years prior to that with a significant decrease in video duration. Also, among videos uploaded in the last five years, the number of videos for patients was significantly higher. Healthcare professionals have become more aware that people are using YouTube for information purposes. In recent years, it has become more possible for people to get quality content with short videos without distracting them.
